# Caesarean delivery and neonatal mortality: evidence from selected slums in and around Dhaka city, Bangladesh- A prospective cohort study

**DOI:** 10.1186/s41043-024-00563-x

**Published:** 2024-05-18

**Authors:** Abdur Razzaque, Razib Chowdhury, AHM Golam Mustafa, Md Arif Billah, Shakera Naima, Sohana Shafique, Bidhan Krishna Sarker, Mohammad Zahirul Islam, Minjoon Kim, Margub Aref Jahangir, Ziaul Matin, Jannatul Ferdous, Maya Vandenent, Anisur Rahman

**Affiliations:** 1 International Centre for Diarrhoeal Disease Research, 68 Shaheed Tajuddin Ahmed Sarani, Mohakhali, Dhaka, 1212 Bangladesh; 2 Embassy of Sweden, Dhaka, Bangladesh; 3 Maternal Newborn Health, UNICEF, New York, USA; 4 UNICEF, Dhaka, Bangladesh; 5 UNICEF, New Delhi, India

**Keywords:** Caesarean delivery, Neonatal mortality, Slum, Health and Demographic Surveillance System, Bangladesh

## Abstract

**Background:**

This study examined the neonatal mortality for newborn of women who delivered by caesarean section or vaginally using a prospective cohort.

**Methods:**

A total of 6,989 live births registered from 2016 to 2018, were followed for neonatal survival from the selected slums of Dhaka (North and South) and Gazipur city corporations, where icddr,b maintained the Health and Demographic Surveillance System (HDSS). Neonatal mortality was compared by maternal and newborn characteristics and mode of delivery using z-test. Logistic regression model performed for neonatal mortality by mode of delivery controlling selected covariates and reported adjusted odd ratios (aOR) with 95% confidence interval (CI).

**Results:**

Out of 6,989 live births registered, 27.7% were caesarean and the rest were vaginal delivery; of these births, 265 neonatal deaths occurred during the follow-up. The neonatal mortality rate was 2.7 times higher (46 vs. 17 per 1,000 births) for vaginal than caesarean delivered. Until 3rd day of life, the mortality rate was very high for both vaginal and caesarean delivered newborn; however, the rate was 24.8 for vaginal and 6.3 per 1,000 live births for caesarean delivered on the 1st day of life. After adjusting the covariates, the odds of neonatal mortality were higher for vaginal than caesarean delivered (aOR: 2.63; 95% CI: 1.82, 3.85). Additionally, the odds were higher for adolescent than elderly adult mother (aOR: 1.60; 95% CI: 1.03, 2.48), for multiple than singleton birth (aOR: 5.40; 95% CI: 2.82, 10.33), for very/moderate (aOR: 5.13; 95% CI: 3.68, 7.15), and late preterm birth (aOR: 1.48; 95% CI: 1.05, 2.08) than term birth; while the odds were lower for girl than boy (aOR: 0.74; 95% CI: 0.58, 0.96), and for 5th wealth quintile than 1st quintile (aOR: 0.59, 95% CI: 0.38, 0.91).

**Conclusion:**

Our study found that caesarean delivered babies had significantly lower neonatal mortality than vaginal delivered. Therefore, a comprehensive delivery and postnatal care for vaginal births needed a special attention for the slum mothers to ensure the reduction of neonatal mortality.

**Supplementary Information:**

The online version contains supplementary material available at 10.1186/s41043-024-00563-x.

## Background

Globally, the neonatal mortality has declined (by 51% from 37 deaths per 1000 live births in 1990 to 18 deaths per 1000 live births in 2021) appreciably over the last few decades [1]. However, the South Asian countries ranked second in terms of neonatal deaths (23 per 1000 live births), following sub-Saharan Africa (27 per 1000 live births) [1]. Bangladesh has also made appreciable progress in declining neonatal mortality in recent decades, and these rates are now 20 per 1,000 live births in 2022, compared to 27 per 1,000 live births in 2017 [2]. However, the rate is still high and concerning for the lower socioeconomic groups, especially for the slum population [3], which can be a hinderance to achieving the sustainable development goals for Bangladesh.

The progress in neonatal mortality is mainly due to the increase in access to comprehensive emergency obstetric care and caesarean sections [4]. In fact, caesarean delivery is a lifesaving procedure when vaginal delivery shows a risk to the mother or baby due to antepartum haemorrhage, foetal distress, an abnormal position of the baby, or hypertensive disease [5]. Over the last few decades, caesarean delivery has increased rapidly all over the world. In 1990, 6.7% of women gave birth by caesarean delivery worldwide, and it increased to 21.1% in 2018, while 23.1% of caesarean deliveries occurred in Asia [6]. Based on the projections, it has been stated that by 2030, 28.5% of women will give birth by caesarean section worldwide, with a wide variation across the regions, with 63.4% in Eastern Asia, and 50% in Western Asia [6, 7]. In Bangladesh, the rate of caesarean delivery also increased from 3% in 1999–2000 to 9% in 2007, 17% in 2011, 23% in 2014, and 33% in 2017-18 [2], and a similar pattern was also found among slum dwellers [8]. Such increases raised the issue of overutilization of caesarean deliveries, that not only pose adverse health effects on mother and child, but also incur burdens for families and health systems [9].

The recommended level of caesarean delivery in a population is 5–15% [10]; however, a figure below 5% indicates that a substantial proportion of women lack access to caesarean delivery, while a figure above 15% suggests overutilization of caesarean delivery [9, 11]. Studies around the world reported conflicting results for those examined for caesarean delivery and subsequent maternal and newborn health outcomes [11–16]. However, the caesarean delivery rate higher than 10–15% was not associated with a decrease in maternal and neonatal mortality rates [14, 16]. Using data from 126 countries, Fahmy, Crispim [17] reported that caesarean delivery was positively associated with maternal, neonatal, and infant mortality in countries where caesarean delivery rates were more than 15%. In fact, Althabe, Sosa [12] found no such relationship in medium- and high-income countries but for low-income countries, when caesarean delivery available for high-risk pregnancies contributed to improving maternal and neonatal outcomes. Moreover, it has also been reported that both preterm delivery and neonatal mortality rates rose with the increase in caesarean deliveries [18]. Caesarean section also increases the chance of having preterm or early term babies [19] and even neonatal deaths [18].

Studies conducted in South Asian countries, including Bangladesh, have raised concern about the high use of caesarean deliveries due to non-regulatory dominance of private health facilities [8, 9, 20]. Moreover, the pregnant women were also afraid of the vaginal delivery pain, and in many aspects, they were also motivated to have a caesarean section by the service providers. Additionally, the growing trend of private healthcare facilities and their profit-making tendencies are also promoting the caesarean delivery, regardless of the long term impact on maternal and neonatal health. These tendencies are found higher among the more educated, wealthier, and urban areas [8, 21–23]. As a consequence, delivery related out-of-pocket expenditure as well as the long-term impact of maternal and neonatal health were often compromised, especially for slum population. However, regarding urban primary healthcare services, the network of pluralistic public health sectors are stronger in rural areas than in urban areas [24].

Though there are many reasons why women were lining up for caesarean delivery over vaginal delivery, in spite of their low socioeconomic conditions. From the socio-demographic perspective, neonatal survival is one of the major causes that has greater importance. Most of the studies about neonatal mortality regarding the mode of delivery were used national-level aggregated data, and there are very limited resources to evaluate the relationship for urban areas, especially for the slum population [8, 21–23]. Moreover, most of the earlier studies used ecological or survey data to establish the association between caesarean delivery and child health outcomes; however, longitudinal data following the birth cohort is extremely limited [13]. Therefore, this study examines the newborn survival up to the neonatal period of those delivered caesarean vs. vaginal and to identify the possible risk factors. Our study used data from selected slums, where urban HDSS has been in full operation since 2016.

## Methodology

### Study design and settings

The data for this study came from selected slums in Dhaka (North and South) and Gazipur City Corporations, where icddr,b has been maintaining a Health and Demographic Surveillance System (HDSS) since 2016 for over 120,000 people. The study area is in close proximity to where people of middle- and high-income groups live and many garment factories; this is an opportunity for the slum dwellers to get easy access to work from home to these places for their livelihood. The people living in urban slums are at risk groups as the environment in the slum is favourable for disease transmission with the overcrowded living conditions and limited access to public health infrastructure [25]. For this study we extracted the data from urban HDSS that contains the date of conception, date of delivery, mode of delivery, number of antenatal visits, litter size, sex of the baby, and data on the mother’s date of birth, education, occupation and a battery list of wealth quintile.

### Participants

**Fig. 1:**
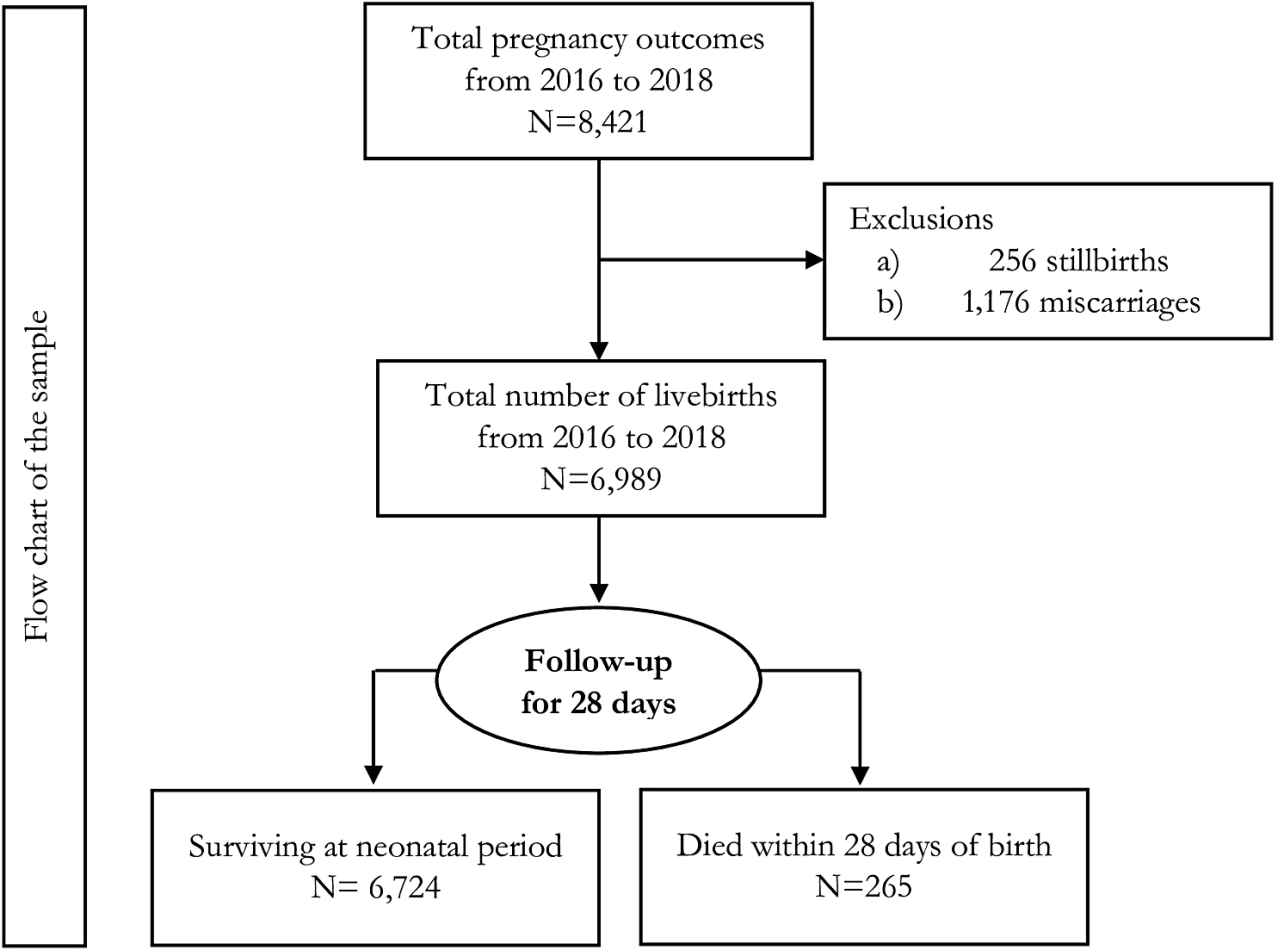
Flow chart of the sample selection of the study

We used a birth cohort who born alive within the study sites from 2016 to 2018. These live births were followed for their survival until the neonatal period (< 29 days). During the study period, a total of 8,421 conceptions had been recorded where 82.99% (*n* = 6,989) were live births, 3.05% (*n* = 256) were stillbirths, and 8.66% (*n* = 729) and 5.31% (*n* = 447) were ended up with miscarriage and abortion, respectively. Of these live births, 265 died during the neonatal period. Details of these were provided in Fig. 1.

### Variables

#### Exposures

The standard clinical and surgical definition of caesarean delivered babies was followed, i.e., a foetal delivery through an open abdominal incision and an incision in the uterus, whereas for vaginal delivery, those babies were delivered through the birth canal with or without instrumental and medicinal support. The vaginal delivery could either be at home or at a facility. For vaginal deliveries, the neonatal survivals were checked for those delivered at home (4.3% died) and those at facilities (5.3% died), and no significant difference in survival was found between these two groups. So, in the analyses, the mode of delivery was categorised into two (vaginally and caesarean delivered).

#### Outcome variable

Our main outcome was neonatal death, which were accounted for as those deaths within 28 days after birth (0 to 28 days of life) and was dichotomized (dead or alive).

#### Covariates

The covariates were selected after reviewing the relevant studies in LMICs that match the HDSS variables. Mother’s age at birth was calculated by subtracting the mother’s date of birth from her child’s date of birth, converted in years, and categorized (< 18, 18–24, and 25 or more years). The gestational age was calculated by subtracting the delivery date and the date of last menstrual period (LMP) of the conception. Preterm birth was defined as a live birth for those born between 28 and 36 weeks of gestation and further categorized as very/moderate preterm (28 to 33 weeks) and late preterm (34 to 36 weeks) births; those born at 37 or more weeks of gestation were classified as term birth. Sex of the child (boy and girl), mother’s years of schooling (0, 1–4 and 5 or more years), litter size (singleton and multiple), and antenatal care visits of the respective pregnancy (0, 1–3, and 4 or more). Mother’s occupation was used for the mother’s working status, for example, women who were economically active and earning for their family were categorised as working, otherwise not working. We also recorded the wealth quintile, which was derived from 15 relative household assets and possessions, such as- chair/table, dining table, bed, chawki, sofa, wardrobe, radio, television, fridge, mobile phone, watch, electric fan, rickshaw/van, computer, and sewing machine; using principle component analyses (PCA). The generated PCA scores were then indexed into five quintiles (1st, 2nd, 3rd, 4th and 5th quintile) [26]. As the study variables were derived from the HDSS cohorts, where the information was collected from respondents using a predefined questionnaire, we had very limited information about the birth histories of the mothers and other clinical variables.

### Quality of data

The urban HDSS has been running quarterly after the baseline census since end of 2015, and the standard protocol was followed to collect data with 17 trained female Field Workers and 3 supervisors. The data was validated by the data management team, and any inconsistencies reported to the supervisors are checked by the Field Workers, who consult available records as well as conduct the field visit if needed. Field supervisors also observed 2–3% of the households to ensure the quality of the data [27]. To minimise reporting error, female Field Workers were adequately trained to collect the data, particularly for date of event (conception, pregnancy outcome, and death); however, reported conception date is usually criticised for accuracy. For ascertaining conception, the female Field Worker asked each eligible married woman (15–49 years) during their routine data collection about whether they had been menstruating or not; if not, then they asked about their last menstruation period to ascertain the conception status. Once the conception was confirmed, the woman was followed for subsequent pregnancy outcomes.

### Statistical analyses

Both bivariate and multivariable approaches were encompassed for the study objective. Chi-square tests were employed to assess the association between neonatal mortality and other covariates with the mode of delivery. These tests are appropriate for analysing the general association of nominal variables, providing insights into the distribution and potential relationships between categorical variables. A proportional z-test (Eq. 1) was utilized to quantify the ratio of neonatal mortality by mode of delivery for maternal and newborn characteristics. The z-test allows for the comparison of proportions between groups, in this case, the proportions of neonatal mortality for vaginal and caesarean deliveries across different clusters of maternal and newborn characteristics.

1 $${z}_{i}=\frac{({p}_{vi}-{p}_{ci})-0}{\sqrt{p\left(1-p\right)(\frac{{n}_{vi}-{n}_{ci})}{{n}_{vi}{n}_{ci}}}}$$

${p}_{vi}$ and ${p}_{ci}$ were the proportions of vaginal (v) and caesarean (c) deaths for each cluster (i); ${n}_{vi}$ and ${n}_{ci}$ were the respective numbers of live births, and $p$ was the overall proportion of deaths.

Day-specific neonatal mortality rates were reported by mode of delivery. This temporal analysis provides a detailed understanding of how neonatal mortality rates vary across different days of the neonatal period. We applied a bivariate logistic model (Model-I) to examine the effects of caesarean delivery on neonatal mortality. Later, all the covariates, such as age of mother at birth, sex of children, mother’s education, mother’s working status, wealth quintile, litter size, number of antenatal visits, and categories of gestational age were added and re-run the model (Model-II). All the reports were interpreted as odds ratios (with and without adjusted by the covariates) and 95% CI.

All the analyses were performed in the STATA 16.1 Windows version (Stata.Corp, TX), and the whole manuscript was reported under the CONSORT guidelines (see the supplementary file).

#### Patient and public involvement

No patients were directly involved in setting the research question, outcome measures, or the design of the study. They were not involved in the interpretation of the results; however, there is a plan to disseminate the results among mothers and women attending health care service centres. Their written consent/assent has been taken before the data collection.

## Results

Out of 6,989 live births, 265 died during the neonatal period, with an average follow-up duration of 27.03 days. The distribution of covariates related to maternal and newborn characteristics by mode of delivery usually differed significantly, except for the age of the mother and the mother’s working status (Table 1). In fact, caesarean delivery usually varied by sex of the child (boys were more likely to be delivered by caesarean than girls), mother’s education (an educated mother delivered more by caesarean than a less educated mother), litter size (multiple births were delivered more by caesarean), number of antenatal visits (those women who had 4 or more antenatal visits were delivered more by caesarean than those who had ‘no’ or ‘1–4’ antenatal visits), and preterm birth (those women who delivered newborn at late preterm had more caesarean delivery than those who delivered at very/moderate preterm and term births).

**Table 1:** Percent distribution of maternal and newborn characteristics by mode of delivery (*N* = 6,989)

Variables	Vaginal % (*n*)	Caesarean % (*n*)	*p*-value	Total % (*n*)
**Age of mother at birth (years)**			*p* = 0.156	
<18	9.0 (456)	7.8 (152)		8.7 (608)
18–24	54.8 (2769)	54.1 (1047)		54.6 (3816)
25 or more	36.2 (1828)	38.1 (737)		36.7 (2565)
**Sex of child**			*p* < 0.001	
Boy	49.9 (2520)	54.7 (1060)		51.2 (3580)
Girl	50.1 (2533)	45.3 (876)		48.8 (3409)
**Mother’s education (years of schooling)**			*p* < 0.001	
None	31.0 (1568)	20.4 (394)		28.1 (1962)
1–4	23.0 (1163)	17.9 (347)		21.6 (1510)
5 or more	46.0 (2322)	61.7 (1195)		50.3 (3517)
**Mother’s working status**			*p* = 0.506	
Not working	73.4 (3711)	74.2 (1437)		73.7 (5148)
Working	26.6 (1342)	25.8 (499)		26.3 (1841)
**Wealth quintile**			*p* < 0.001	
1st quintile	24.3 (1227)	14.8 (286)		21.6 (1513)
2nd quintile	18.9 (953)	12.8 (247)		17.2 (1200)
3rd quintile	22.0 (1112)	18.9 (366)		21.2 (1478)
4th quintile	18.7 (945)	21.7 (421)		19.5 (1366)
5th quintile	16.1 (816)	31.8 (616)		20.5 (1432)
**Litter size**			*p* < 0.001	
Single	99.3 (5019)	97.5 (1888)		98.8 (6907)
Multiple	0.7 (34)	2.5 (48)		1.2 (82)
**No. of antenatal visits**			*p* < 0.001	
0	18.8 (951)	8.6 (166)		16.0 (1117)
1–3	51.3 (2591)	36.7 (710)		47.2 (3301)
4 or more	29.9 (1511)	54.7 (1060)		36.8 (2571)
**Gestational age (weeks)**			*p* < 0.05	
≤33	6.0 (303)	6.0 (116)		6.0 (419)
34–36	15.0 (758)	17.5 (339)		15.7 (1097)
37 or more	79.0 (3992)	76.5 (1481)		78.3 (5473)
**Neonatal deaths**			*p* < 0.001	
No	95.4 (4821)	98.3 (1903)		96.2 (6724)
Yes	4.6 (232)	1.7 (33)		3.8 (265)
**Total**	**72.3 (5053)**	**27.7 (1936)**		**100.0 (6989)**

*Note* Very/moderate preterm = ≤ 33 weeks, Late preterm = 34–36 weeks, Term birth = 37 or more weeks

Out of 5,053 live births by vaginal delivery, 232 died, while out of 1,936 live births by caesarean delivery, 33 died, resulting in 46.9 and 17.0 neonatal mortality (per 1,000 live births), respectively (Table 2). The mortality ratios (vaginal vs. caesarean) by covariates varied between 1.46 and 5.65, with the lowest ratio for those who had no antenatal visit (1.46), and the highest ratio for those who gave birth to multiple babies (5.65). Comparing the neonatal mortality rate for vaginal and caesarean delivery for each of the covariates (Table 3), the overall neonatal mortality rate for vaginal delivery was significantly higher than for caesarean delivery. The proportional differences in neonatal mortality rates for vaginal and caesarean were also significant by clusters of different covariates, except for adolescent mothers, the 2nd wealth quintile, and women who visited no antenatal care service. A detailed set of results was provided in the supplementary table file (see supplementary Table 1).

**Table 2:** Neonatal mortality rate (per 1,000 live births) by maternal and neonatal characteristics for vaginal and caesarean delivery

Variables	Vaginal: Mortality rate (no. of live births)	Caesarean: Mortality rate (no. of live births)	Ratio: Vaginal vs. Caesarean
**Age of mother at birth (years)**			
< 18	61.4 (456)	26.3 (152)	2.33
18–24	48.0 (2769)	16.2 (1047)	2.96***
25 or more	38.8 (1828)	16.2 (737)	2.39 **
**Sex of child**			
Boy	53.9 (2520)	16.0 (1060)	3.37***
Girl	37.8 (2533)	18.2 (876)	2.08***
**Education of women (years of schooling)**			
None	48.4 (1568)	22.8 (394)	2.12*
1–4	49.8 (1163)	17.2 (347)	2,89**
5+	42.2 (2322)	15.0 (1195)	2.81***
**Mother’s working status**			
Not working	47.4 (3711)	15.3 (1437)	3.10***
Working	41.7 (1342)	22.0 (499)	1.89*
**Wealth quintile**			
1st quintile	54.6 (1227)	24.5 (286)	2.23*
2nd quintile	48.3 (953)	24.3 (247)	1.99
3rd quintile	43.2 (1112)	16.4 (366)	2.63*
4th quintile	44.4 (945)	19.0 (421)	2.34*
5th quintile	35.5 (816)	9.7 (616)	3.66**
**Litter size**			
Single	43.8 (5019)	15.8 (1888)	2.77***
Multiple	352.9 (34)	62.5 (48)	5.65**
**No. of antenatal visits**			
0	44.1 (951)	30.1 (166)	1.46
1–3	50.9 (2591)	25.3 (710)	2.01**
4 or more	38.3 (1511)	9.4 (1060)	4.07***
**Gestational age (weeks)**			
≤ 33	168.0 (303)	78.0 (116)	2.15***
34–36	50.0 (757)	21.0 (339)	2.38*
37 or more	36.0 (3992)	11.0 (1481)	3.27***
**All**	**45.9 (5053)**	**17.0 (1936)**	2.70***

*Note* *p < 0.05, **p < 0.01, and ***p < 0.001

**Fig. 2:**
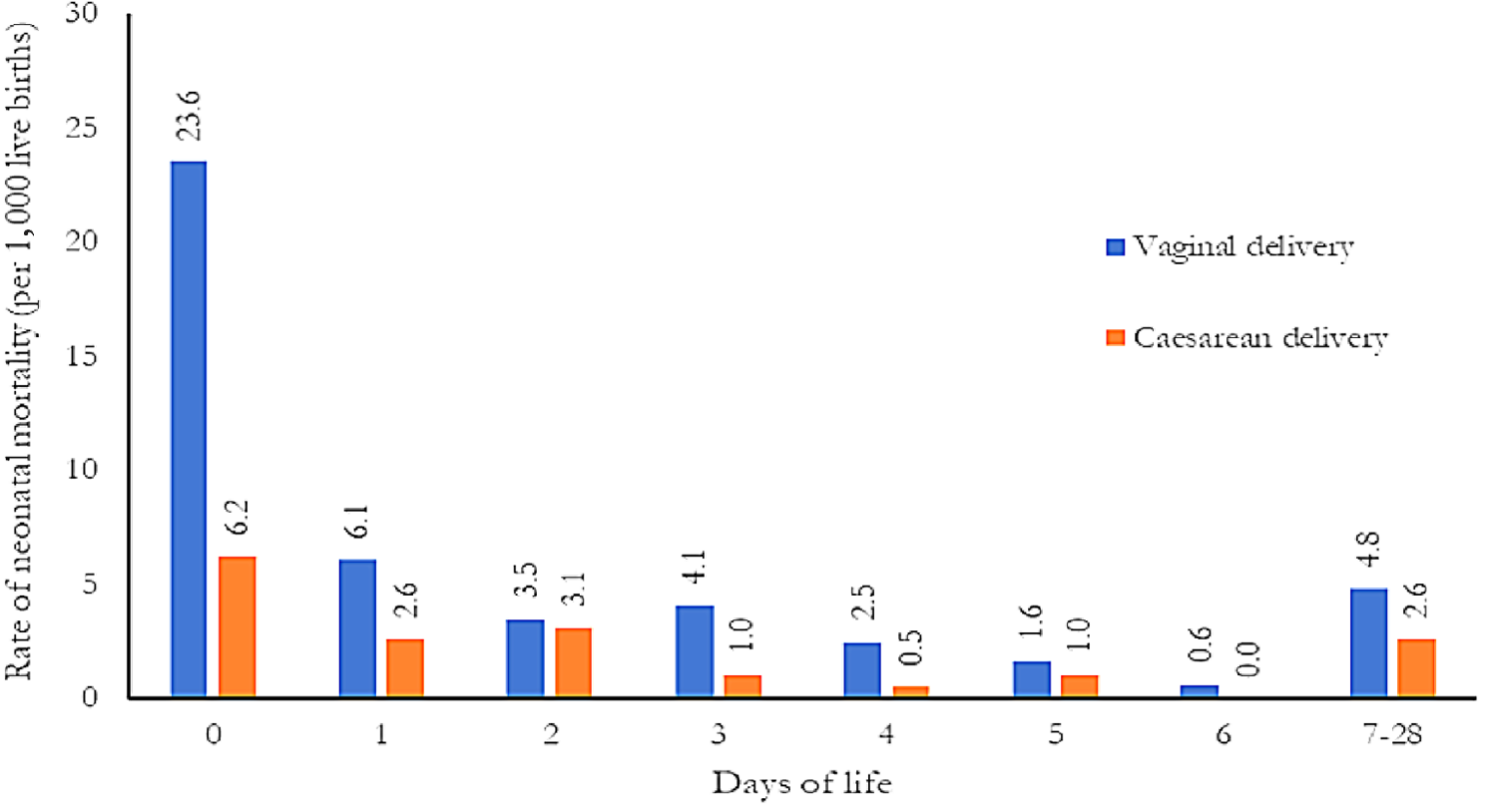
Rate of neonatal mortality per 1,000 live births by age at death and mode of delivery

The neonatal mortality rates were very high until the 3rd day of life for both vaginal and caesarean delivered babies (Fig. 2); however, the rate was exceptionally high (23.6 vs. 6.2 for vaginal vs. caesarean delivery) on the day of birth for vaginal delivery; the mortality differences continued until the late neonatal period (4.8 vs. 2.6 for vaginal vs. caesarean delivery). Detailed day-specific mortality rates were also provided in the supplementary table file (see supplementary Table 2).

In the bivariate regression model (Model-I), the odds of neonatal mortality was higher for vaginal than caesarean delivered (OR: 2.77, 95% CI: 1.92, 4.01) babies. After adjusting the covariates (Model-II), the odds of neonatal mortality were also higher for vaginal than caesarean delivered (aOR: 2.63; 95% CI: 1.82, 3.85) babies. While for the other covariates, we found that the odds of neonatal mortality were higher for the adolescent than the elderly adult mother (aOR: 1.60; 95% CI: 1.03, 2.48), lower for the girl than the boy (aOR: 0.74; 95% CI: 0.58, 0.96), lower for the 5th wealth quintile than the 1st quintile (aOR: 0.59, 95% CI: 0.38, 0.91), higher for multiple than singleton birth (aOR: 5.40; 95% CI: 2.82, 10.33), higher for very/moderate (aOR: 5.13; 95% CI: 3.68, 7.15), and late preterm birth (aOR: 1.48; 95% CI: 1.05, 2.08) than term birth.

**Table 3:** Logistic regression estimates of neonatal mortality (*N* = 6,989)

Factors	Model-I: Odd ratios (95% CI)	*p*-value	Model-II: Odd ratios (95% CI)	*p*-value
**Mode of delivery**				
Caesarean (rc = Vaginal)	0.36 (0.25, 0.52)	*p* < 0.001	0.38 (0.26, 0.55)	*p* < 0.001
**Age of mother at birth (years)**				
< 18 (rc = 25 or more)			1.60 (1.03, 2.48)	*p* < 0.05
18–24 (rc = 25 or more)			1.21 (0.91, 1.62)	*p* = 0.178
**Sex of child**				
Girl (rc = Boy)			0.74 (0.58, 0.96)	*p* < 0.05
**Education of women (years of schooling)**				
1–4 (rc = None)			0.99 (0.70, 1.39)	*p* = 0.948
5 or more (rc = None)			0.81 (0.60, 1.10)	*p* = 0.184
**Mother’s working status**				
Not working (rc = Working)			1.03 (0.76, 1.38)	*p* = 0.864
**Wealth quintile**				
2nd quintile (rc = 1st quintile)			0.82 (0.56, 1.19)	*p* = 0.293
3rd quintile (rc = 1st quintile)			0.75 (0.52, 1.08)	*p* = 0.120
4th quintile (rc = 1st quintile)			0.83 (0.57, 1.21)	*p* = 0.329
5th quintile (rc = 1st quintile)			0.59 (0.38, 0.91)	*p* < 0.05
**Litter size**				
Multiple (rc = Single)			5.40 (2.82, 10.33)	*p* < 0.001
**No. of antenatal visits**				
1–3 (rc = None)			1.18 (0.83, 1.66)	*p* = 0.356
4 or more (rc = None)			0.84 (0.56, 1.25)	*p* = 0.392
**Gestational age (weeks)**				
≤ 33 (rc = 37 or more)			5.13 (3.68, 7.15)	*p* < 0.001
34–36 (rc = 37 or more)			1.48 (1.05, 2.08)	*p* < 0.05
**Constant**	0.05 (0.04, 0.06)	*p* < 0.001	0.04 (0.03, 0.07)	*p* < 0.001
-2 Loglikelihood (df)	1108.4715 (1)		1040.4135 (16)	
LR chi2 (p-value)	37.23 *(p < 0.001)*		173.35 (*p < 0.001)*	
Pseudo R^2^	0.0165		0.0769	

rc = Reference category; df = degrees of freedom; LR = Likelihood ratio

*Note* Very/moderate preterm = ≤ 33 weeks, Late preterm = 34–36 weeks, Term birth = 37 or more weeks

Model-I: mode of delivery; Model-II: mode of delivery along with selected covariates

## Discussion

The study reported that 27.7% of births were caesarean delivered, and these births usually varied significantly by maternal and newborn characteristics. The overall neonatal mortality rate of our study was estimated at 38 per 1,000 live births, while the rate of vaginal delivery was significantly higher than caesarean delivery. The mortality rate was very high for both caesarean and vaginal deliveries up to the 3rd day of life, and the rates were consistently higher for vaginal deliveries. However, on the first day of life, the mortality rate for vaginal deliveries (23.6 per 1,000 live births) was higher than for caesarean deliveries (6.2 per 1,000 live births). We also found that the odds of neonatal mortality were much lower for caesarean delivery for both unadjusted and after adjusting covariates. Moreover, the covariates, such as for adolescent mothers, multiple birth, very/moderate, and late preterm birth, have higher odds of neonatal mortality. While giving birth to girl babies and women from the 5th wealth quintile had fewer odds of neonatal mortality.

More than one out of four slum women were going for caesarean delivery, which is consistent with one of the previous studies conducted for the slum population [8]. A recent urban health survey also reported that about 31.3% of all births in slum areas were caesarean deliveries [28] However, the prevalence is comparatively much lower than the national estimates [21, 23], while the estimate is close to the national rural caesarean prevalence. It is worth noting that the slum dwellers are also migrants from the rural area who came here to find work for their livelihood. Other studies in Bangladesh found that caesarean delivery varied significantly by maternal and newborn characteristics, with a higher caesarean delivery among educated mothers, boys, multiple births, those who had more antenatal visits, and preterm births compared to comparable groups [8, 29].

We found similar estimates of urban neonatal mortality reported in the Bangladesh demographic health survey 2017-18 [2]; however, it was higher than the rates reported for the slum population in the Bangladesh urban health survey 2021 [28]. Our study reported that neonatal mortality was significantly higher for vaginal than caesarean delivered newborns; the pattern remains the same after being adjusted for maternal and newborn characteristics. Similar findings were found in the MANOSHI project conducted in urban slums, where 85% of neonatal deaths occurred during vaginal delivery [30]. While in India, Gondwe, Betha [13] found that caesarean delivery significantly reduced neonatal mortality. Other studies also found that neonatal mortality was higher for vaginal births than for preterm, term, or complicated births [31–33]. Moreover, the neonatal mortality rate by caesarean delivery is significantly low for all socioeconomic groups, except for adolescent mothers, mothers from the lower wealth quintile, and mothers who got no ANC. Therefore, caesarean delivery is an immediate life-saving intervention for neonatal health, which is also true for the slum population.

In terms of other covariates, our study also found that very preterm and moderate preterm births significantly increased neonatal mortality after controlling for the other variables. This is also true for developed countries; for example, in Switzerland, the incidence of neonatal mortality increases for lower gestational ages, while the mortality is lower for term births, irrespective of mode of delivery [34]. Moreover, the adolescent mothers or twin pregnancies were at risk of any adverse pregnancy-related outcomes and increased neonatal mortality [35–39], which is also aligned with our study findings. For the slum population, their socioeconomic and cultural factors also contribute to these deaths for at risk mothers [3, 40]. However, for richer women and for the birth of girls, the odds of neonatal mortality are lower. The mothers from well-off households had more access to healthcare facilities, and socio-economically, they gained advantages after conception [41]. In the slum area, the well-off families are also more concerned about their healthcare utilization, especially for maternal services [40]. Regarding the sex of the neonates, boys are more vulnerable than girls due to ambient stressors during foetal development, and they have a lesser survival capacity [42–44]. However, the low quality of health care seeking is more prominent for girls’ children in South Asian as well as Bangladeshi cultures [42, 44, 45].

Our study reported that the death rate was high until the 3rd day of life for both vaginal and caesarean deliveries; however, there was an exceptionally high mortality on the 1st day of life for those vaginal deliveries. A systematic review pooled 22 studies and found that around 57% of the neonatal deaths occurred during the first 3 days of life, as neonatal mortality due to asphyxia, prematurity, and malformations were mostly caused during these first 3 days of life [46]. These high rates, along with our study, demonstrate the lack of evidence-based interventions beginning in the antenatal period, which is relatively low among urban slum mothers [46–48]. Relatively lower mortality for caesarean delivery than vaginal on the 1st day of life could be due to the care received by the mother and newborn from the hospital, as the caesarean-delivered mother had to stay for a longer time at the hospital [2, 49–52]. On the other hand, for those vaginal deliveries at the hospital, about 50% of mothers are released within 24 h after the delivery [2]; however, few of the vaginal deliveries (mother or newborn), especially in slums received postnatal care services [26, 49]. As a result, quality delivery and postnatal care services are important for the mother and neonates to improve their survival. In this regard, WHO and UNICEF jointly stated that the promotion of universal access to antenatal care, skilled birth attendance, and early postnatal care would contribute to a sustained reduction in maternal and neonatal mortality [53].

### Strengths and limitations of this study

This study has many strengths and a few limitations. Firstly, in the study, slums represent the majority of the slum population of the city, and they are the poorest section of the rural area that migrated to the city for a better livelihood. So, the findings of the study can be generalized for the urban poor. Besides, we used the birth cohort data that followed prospectively for neonatal survival with the rigorous methodology of active surveillance. Therefore, the identified risk factors appropriately demonstrated their effects on neonatal survival. However, due to data limitations, the model has the limitation of considering several reproductive and pregnancy histories, such as birth order, previous child deaths, and complications of pregnancy and delivery. Besides, the LMP of women were not collected using any of the clinical information of the mother; therefore, there is a limited but possible chance of recall bias in gestational age calculation; however, it has a less likely chance to alter the effect sizes. Moreover, this study only focused on the effects of the mode of delivery on neonatal survival.

## Conclusions

Our study reported that caesarean delivered babies had significantly lower neonatal mortality than those delivered vaginally, which is consistent across the different socioeconomic groups. Moreover, the first 3 days of neonatal life are very crucial for neonatal survival. In summary, the study highlights the complex interplay of factors influencing neonatal mortality among the urban slum population, with mode of delivery playing a significant role. Therefore, addressing neonatal mortality requires a comprehensive medical intervention approach, especially for vaginal births along with the broader factors related to maternal and newborn characteristics. Moreover, the researchers and healthcare providers can use this information to inform strategies and interventions aimed at reducing neonatal mortality, considering the identified risk factors, where a special attention should be given to the vaginal delivery in achieving the Sustainable Development Goal 3 for Bangladesh.

### Electronic supplementary material

Below is the link to the electronic supplementary material.

Supplementary Material 1

Supplementary Material 2

## Declarations

### Provenance and peer review

Not commissioned; externally peer reviewed.

### Data sharing statement

The data sets have been generated and analysed during the current study, and they are not publicly available due to the organization policy but are available from the corresponding author on reasonable request. For additional information please refer to http://www.icddrb.org/policies

## Abbreviations

aOR: Adjusted Odd Ratio
CI: Confidence Interval
df: Degrees of Freedom
HDSS: Health and Demographic Surveillance System
LMP: Last Menstrual Period
NGOs: Non-Government Organizations
PCA: Principal Component Analysis
rc: Reference Category
UNICEF: United Nations Children’s Fund
WHO: World Health Organization
