# Supplementary material for: Caesarean delivery and neonatal mortality: evidence from selected slums in and around Dhaka city, Bangladesh- A prospective cohort study
Source: J Health Popul Nutr. 2024 May 18;43:69. doi: 10.1186/s41043-024-00563-x (PMC11102622; doi:10.1186/s41043-024-00563-x)
Supplement: Supplementary file 1 — Supplementary Material 1 [file 41043_2024_563_MOESM1_ESM.docx]

**Supplementary file**

Supplementary table 1. Two-tailed z-score and p-values of the proportion of neonatal mortality rates by vaginally and caesarean deliveries

| **Variables** | **Mortality rate (no. of livebirths)** | | **z-score** | **p-value** |
| --- | --- | --- | --- | --- |
|  | **Vaginal** | **Caesarean** |  |  |
| **Age of mother at birth (years)** |  |  |  |  |
| <18 | 61.4(456) | 26.3(152) | 1.6784 | p=0.093 |
| 18-24 | 48.0(2769) | 16.2(1047) | 4.5123 | p<0.001 |
| 25 or more | 38.8(1828) | 16.2(737) | 2.9294 | p<0.01 |
| **Sex of child** |  |  |  |  |
| Boy | 53.9(2520) | 16.0(1060) | 5.1218 | p<0.001 |
| Girl | 37.8(2533) | 18.2(876) | 2.809 | p<0.001 |
| **Education of women (years of schooling)** |  |  |  |  |
| None | 48.4(1568) | 22.8(394) | 2.2329 | p<0.05 |
| 1-4 | 49.8(1163) | 17.2(347) | 2.6488 | p<0.01 |
| 5+ | 42.2(2322) | 15.0(1195) | 4.2795 | p<0.001 |
| **Mother’s working status** |  |  |  |  |
| Not working | 47.4(3711) | 15.3(1437) | 5.3738 | p<0.001 |
| Working | 41.7(1342) | 22.0(499) | 2.0072 | p<0.05 |
| **Wealth quintile** |  |  |  |  |
| 1^st^ quintile | 54.6(1227) | 24.5(286) | 2.1254 | p<0.05 |
| 2^nd^ quintile | 48.3(953) | 24.3(247) | 1.6504 | p=0.099 |
| 3^rd^ quintile | 43.2(1112) | 16.4(366) | 2.3695 | p<0.05 |
| 4^th^ quintile | 44.4(945) | 19.0(421) | 2.3093 | p<0.05 |
| 5^th^ quintile | 35.5(816) | 9.7(616) | 3.1328 | p<0.01 |
| **Litter size** |  |  |  |  |
| Single | 43.8(5019) | 15.8(1888) | 5.5563 | p<0.001 |
| Multiple | 352.9(34) | 62.5(48) | 3.3512 | p<0.01 |
| **No. of antenatal visits** |  |  |  |  |
| 0 | 44.1(951) | 30.1(166) | 0.8295 | p=0.407 |
| 1-3 | 50.9(2591) | 25.3(710) | 2.9032 | p<0.01 |
| 4 or more | 38.3(1511) | 9.4(1060) | 4.5005 | p<0.001 |
| **Gestational age (weeks)** |  |  |  |  |
| ≤33 | 168.0(303) | 78.0(116) | 4.4573 | p<0.001 |
| 34-36 | 50.0(757) | 21.0(339) | 2.2371 | p<0.05 |
| 37 or more | 36.0(3992) | 11.0(1481) | 4.8774 | p<0.001 |
| **All** | **45.9(5053)** | **17.0(1936)** | **5.6626** | **p<0.001** |

Supplementary table 2. Detailed neonatal mortality rates by the days of life

| **Days** | **No. of deaths** | | | **No. of live births** | | | **Mortality rate** | | |
| --- | --- | --- | --- | --- | --- | --- | --- | --- | --- |
|  | **Vaginal** | **Caesarean** | **Total** | **Vaginal** | **Caesarean** | **Total** | **Vaginal** | **Caesarean** | **Total** |
| ***0*** | 119 | 12 | 131 | 5,053 | 1,936 | 6,989 | 23.55 | 6.20 | 18.74 |
| ***1*** | 30 | 5 | 35 | 4,934 | 1,924 | 6,858 | 6.08 | 2.60 | 5.10 |
| ***2*** | 17 | 6 | 23 | 4,904 | 1,919 | 6,823 | 3.47 | 3.13 | 3.37 |
| ***3*** | 20 | 2 | 22 | 4,887 | 1,913 | 6,800 | 4.09 | 1.05 | 3.24 |
| ***4*** | 12 | 1 | 13 | 4,867 | 1,911 | 6,778 | 2.47 | 0.52 | 1.92 |
| ***5*** | 8 | 2 | 10 | 4,855 | 1,910 | 6,765 | 1.65 | 1.05 | 1.48 |
| ***6*** | 3 | 0 | 3 | 4,847 | 1,908 | 6,755 | 0.62 | 0.00 | 0.44 |
| 7 | 6 | 1 | 7 | 4,844 | 1,908 | 6,752 | 1.24 | 0.52 | 1.04 |
| 8 | 1 | 0 | 1 | 4,838 | 1,907 | 6,745 | 0.21 | 0.00 | 0.15 |
| 10 | 3 | 1 | 4 | 4,837 | 1,907 | 6,744 | 0.62 | 0.52 | 0.59 |
| 11 | 2 | 1 | 3 | 4,834 | 1,906 | 6,740 | 0.41 | 0.52 | 0.45 |
| 12 | 4 | 0 | 4 | 4,832 | 1,905 | 6,737 | 0.83 | 0.00 | 0.59 |
| 14 | 0 | 1 | 1 | 4,828 | 1,905 | 6,733 | 0.00 | 0.52 | 0.15 |
| 15 | 0 | 1 | 1 | 4,828 | 1,904 | 6,732 | 0.00 | 0.53 | 0.15 |
| 17 | 2 | 0 | 2 | 4,828 | 1,903 | 6,731 | 0.41 | 0.00 | 0.30 |
| 22 | 1 | 0 | 1 | 4,826 | 1,903 | 6,729 | 0.21 | 0.00 | 0.15 |
| 25 | 1 | 0 | 1 | 4,825 | 1,903 | 6,728 | 0.21 | 0.00 | 0.15 |
| 27 | 1 | 0 | 1 | 4,824 | 1,903 | 6,727 | 0.21 | 0.00 | 0.15 |
| 28 | 2 | 0 | 2 | 4,823 | 1,903 | 6,726 | 0.41 | 0.00 | 0.30 |
| ***7-28 days*** | 23 | 5 | 28 |  |  |  | 4.76 | 2.62 | 4.15 |
| **Total** | **232** | **33** | **265** |  |  |  | 46.68 | 17.16 | 38.45 |
| **NMR_d_^*^** | **45.9** | **17.0** | **37.9** |  |  |  |  |  |  |

*NMR_d_ is direct neonatal mortality was estimated by number of total deaths for vaginally, caesarean and overall deliveries divided by the total number of respective births.
