# Supplementary material for: Caesarean delivery and neonatal mortality: evidence from selected slums in and around Dhaka city, Bangladesh- A prospective cohort study
Source: J Health Popul Nutr. 2024 May 18;43:69. doi: 10.1186/s41043-024-00563-x (PMC11102622; doi:10.1186/s41043-024-00563-x)
Supplement: Supplementary file 2 — Supplementary Material 2 [file 41043_2024_563_MOESM2_ESM.docx]

**SUPPLEMENTARY FILE 1.**

STROBE Statement—Checklist of items that should be included in reports of ***cohort studies***

|  | Item No | Recommendation | Check | Page no. | Relevant texts from manuscript |
| --- | --- | --- | --- | --- | --- |
| **Title and abstract** | 1 | (*a*) Indicate the study’s design with a commonly used term in the title or the abstract | √ | 1 & 2 | A prospective cohort study |
|  |  | (*b*) Provide in the abstract an informative and balanced summary of what was done and what was found | √ | 2 | See abstract |
| Introduction | | |  |  |  |
| Background/rationale | 2 | Explain the scientific background and rationale for the investigation being reported | √ | 3-5 | Studies around the world reported conflicting results for those examined for caesarean delivery and subsequent maternal and child health outcomes (11-16). Based on an ecological study, Ye, Zhang (16) reported that caesarean delivery rate higher than 10% was not associated with the decrease in maternal and neonatal mortality rates. Using data from 126 countries, Fahmy, Crispim (17) reported that caesarean delivery was positively associated with maternal, neonatal, and infant mortality in countries where caesarean delivery rates were more than 15%..............  Over the last few decades, caesarean delivery has increased rapidly all over the world. In 1990, 6.7% of women gave birth by caesarean delivery worldwide, and it increased to 21.1% in 2018 with 9.2% in Africa, 23.1% in Asia, 25.7% in Europe, and 39.3% in America (6). …………..  In Bangladesh, the rate of caesarean delivery increased from 3% in 1999-2000 to 9% in 2007, 17% in 2011, 23% in 2014, and 33% in 2017-18 (2)…………… |
| Objectives | 3 | State specific objectives, including any prespecified hypotheses | √ | 6 | ………….. Our study used data from selected slums in and around Dhaka city, where HDSS has been in operation since 2015. |
| Methods | | |  |  |  |
| Study design | 4 | Present key elements of study design early in the paper | √ | 6 | A birth cohort who were born in the study sites from 2016 to 2018 were used in this study. These births were followed for their survival until the neonatal period (<29 days)………. |
| Setting | 5 | Describe the setting, locations, and relevant dates, including periods of recruitment, exposure, follow-up, and data collection | √ | 6-7 | The data for the study came from selected slums in Dhaka (North & South) and Gazipur City Corporations, where icddr,b has been maintaining a Health and Demographic Surveillance System (HDSS) since 2015 for over 120,000 people. A birth cohort who were born in the study sites from 2016 to 2018 were used in this study. These births were……….  The study area is in proximity where people of middle- and high-income groups live and many garment factories; this is an opportunity for the slum…………. one-third had access to sanitary latrine flush to sewerage/septic tank. Sharing of water sources (92%), latrines (90%), and cooking places (60%) were very common in these slums. |
| Participants | 6 | (*a*) Give the eligibility criteria, and the sources and methods of selection of participants. Describe methods of follow-up | √ | 7 | During the study period (2016-2018), 8,421 conception had been recorded where 6,989 were recorded as live births that used as birth cohort for this study. Of these live births, 265 died during the neonatal period. |
|  |  | (*b*) For matched studies, give matching criteria and number of exposed and unexposed | NA |  |  |
| Variables | 7 | Clearly define all outcomes, exposures, predictors, potential confounders, and effect modifiers. Give diagnostic criteria, if applicable | √ | 7-8 | *Exposures:* Mother’s age at birth was calculated by subtracting mother’s date of birth from her child’s date of birth, converted in years and categorized (<18, 18-24, and 25 or more years). Gestation age was calculated by subtracting delivery date and date of conception. Preterm birth was defined as a livebirth those born between 28 and 36 weeks of gestation and further categorized as very/moderate preterm (28 to 33 weeks) and late preterm (34 to 36 weeks) births; those born at 37 or more weeks of gestation were classified as term birth. Sex of the child (boy and girl), mother’s years of schooling (0, 1-4 and 5 or more years), mother’s working status (working and not working), litter size (singleton and multiple), antenatal care visits during pregnancy period (0, 1-3, and 4 or more). Later the mother’s occupation was converted into mother’s working status who were economically active and earn for their family.  The standard clinical and surgical definition of caesarean delivered babies were followed, whereas, for the vaginal delivery, those babies delivered through birth canal with or without instrumental and medicinal support. The vaginal delivery could either be at home or at facility. For vaginal delivered, the neonatal survivals were checked for those delivered at home (4.3% died) and those at facility (5.3% died) and found no significant difference in survival of these two groups. So, in the analyses the mode of delivery was categorised into two (vaginal and caesarean).  *Outcome variable:* Our main outcome, neonatal deaths were accounted to those deaths within 28 days after births (0 to 28 days of life) and was dichotomized (death or alive). |
| Data sources/ measurement | 8* | For each variable of interest, give sources of data and details of methods of assessment (measurement). Describe comparability of assessment methods if there is more than one group | *√* | 7-8 | See above (item no 7) |
| Bias | 9 | Describe any efforts to address potential sources of bias |  | 8-9 | The urban HDSS running since 2015, the standard protocol was followed to collect data with 17 trained female Field Workers with 3 supervisors. The data was validated by the data-management team and any inconsistency reported to the supervisors are checked by the Field Workers consult available records, as well as through field visit, if needed. Field supervisors also visited 2-3% of the households for ensure the data quality (27). Therefore, no sampling error as each household is covered, however there could be reporting bias. To minimise reporting error, female Field Workers were adequately trained to collect the data particularly for date of event (conception, pregnancy outcome, and death), however, reported conception date is usually been criticised for accuracy. For ascertaining conception, the female Field Worker asked each eligible married woman (15-49 years) during their routine data collection about whether they had been menstruating or not; if not, then they asked about their last mensuration period to ascertain the conception status. Once the conception was confirmed, the woman was followed for subsequent pregnancy outcomes. The death data contains information on the date of death and cause of death; these deaths were collected from the informed household member. The interviewer then wrote down a brief description of the cause of death; however, cause of death data has limitations as it is not collected through structured questionnaire. Subsequently, the death data were coded as the cause by a trained medical-assistant under the guidance of a physician. |
| Study size | 10 | Explain how the study size was arrived at | √ | 7 | During the study period (2016-2018), 8,421 conception had been recorded where 6,989 were recorded as live births that used as birth cohort for this study. Of these live births, 265 died during the neonatal period. |
| Quantitative variables | 11 | Explain how quantitative variables were handled in the analyses. If applicable, describe which groupings were chosen and why | √ | 6-7 | See above (item no 7) |
| Statistical methods | 12 | (*a*) Describe all statistical methods, including those used to control for confounding | √ | 9 | Both bivariate and multivariate analyses were performed. Chi-square tests were used for a general association of nominal variables. For multivariate analyses, multiple logistic regression analysis was performed to examine the effects of caesarean delivery on neonatal mortality; odds ratios and 95% confidence intervals were calculated. For the adjusted regression model, age of mother, sex of children, mother’s education, mother’s working status, litter size, mode of delivery, number of antenatal visits, and categories of gestation age (preterm birth) were treated as independent variables. |
|  |  | (*b*) Describe any methods used to examine subgroups and interactions | √ | 9 | For the adjusted regression model, age of mother, sex of children, mother’s education, mother’s working status, litter size, mode of delivery, number of antenatal visits, and categories of gestation age (preterm birth) were treated as independent variables. |
|  |  | (*c*) Explain how missing data were addressed | NA |  |  |
|  |  | (*d*) If applicable, explain how loss to follow-up was addressed | NA |  |  |
|  |  | (*e*) Describe any sensitivity analyses | NA |  |  |
| Results | | |  |  |  |
| Participants | 13* | (a) Report numbers of individuals at each stage of study—eg numbers potentially eligible, examined for eligibility, confirmed eligible, included in the study, completing follow-up, and analysed | √ | 9 | Out of 6,989 births, 265 died during the neonatal period, with average follow-up duration of 27.03 days. |
|  |  | (b) Give reasons for non-participation at each stage | NA |  |  |
|  |  | (c) Consider use of a flow diagram | NA |  |  |
| Descriptive data | 14* | (a) Give characteristics of study participants (eg demographic, clinical, social) and information on exposures and potential confounders | √ | 9-10 | The distribution of maternal and new-born’s characteristics by mode of delivery usually differed significantly, except for age of mother and mother’s working status (Table 1). In fact, caesarean delivery usually varied by sex of the child (boys were more likely to be delivered by caesarean than girls), mother’s education (educated mother…………….. |
|  |  | (b) Indicate number of participants with missing data for each variable of interest | NA |  |  |
|  |  | (c) Summarise follow-up time (eg, average and total amount) | √ | 9 | ………..with average follow-up duration of 27.03 days |
| Outcome data | 15* | Report numbers of outcome events or summary measures over time | √ | 13 page and figure 1 | The risk of death for new-borns showed that the risk was very high until the 3^rd^ day of life for both vaginal and caesarean delivered new-born (Figure 1); however, the risk of death was exceptionally high (3.9 times) on the day of birth for vaginal delivery; the mortality differences continued until the late neonatal period (1.8 times). |
| Main results | 16 | (*a*) Give unadjusted estimates and, if applicable, confounder-adjusted estimates and their precision (eg, 95% confidence interval). Make clear which confounders were adjusted for and why they were included | √ | 14-15 and table 3 | In the regression analysis, the risk of neonatal mortality was higher for vaginal than caesarean delivered (aOR= 3.01; 95% CI: 1.80, 5.04), higher for adolescent than elderly adult mother (aOR=1.64; 95% CI: 1.06, 2.55), lower for girl than boy (aOR=0.75; 95% CI: 0.58, 0.96), lower for mother who had 5 or more years of schooling than those who had no schooling (aOR=0.77; 95% CI: 0.57, 1.03), higher for multiple than singleton birth (aOR=5.50; 95….. |
|  |  | (*b*) Report category boundaries when continuous variables were categorized | NR |  |  |
|  |  | (*c*) If relevant, consider translating estimates of relative risk into absolute risk for a meaningful time period | NA |  |  |
| Other analyses | 17 | Report other analyses done—eg analyses of subgroups and interactions, and sensitivity analyses | √ | 11-12 and table 2;  And 15-16 with table 4 | Out of 5,053 births by vaginal delivery, 232 died, while out of 1,936 births by caesarean delivery, 33 died, resulting in 46.0 and 17.0 neonatal mortality rates (per 1,000 births) respectively; 2.7 times neonatal mortality for vaginal than caesarean delivered (Table 2). Comparing the neonatal mortality for vaginal and caesarean delivery for each of the maternal and new-born characteristics (Table 2), the rates were significantly higher for vaginal delivery than for caesarean……………  The causes of death differ for those vaginal and caesarean delivered (Table 4). For those vaginal-delivered, the most common causes of death were delivery complications (34.5%), followed by pneumonia/respiratory infections (25.9%), and premature births (7.7%); the patter remain same for those hospital and those……… |
| Discussion | | |  |  |  |
| Key results | 18 | Summarise key results with reference to study objectives | √ | 16 | The study reported that 27.7% of births were caesarean delivered and these births usually varied significantly by maternal and new-born characteristics. The overall neonatal mortality of vaginal delivery was 2.7 times higher (46.0 vs 17.0) than caesarean delivered. The risk of death was very high for both caesarean and vaginal delivered up to the 3^rd^ day of life; however, on the 1^st^ day of life, the risk of death was 3.8 times higher (24.8 vs 6.3) for vaginal than for caesarean delivered…………… |
| Limitations | 19 | Discuss limitations of the study, taking into account sources of potential bias or imprecision. Discuss both direction and magnitude of any potential bias | √ | 18-19 | Limitation of the study is that the data came from selected slums (more stable one) and didn’t include the vulnerable one (side of rail line); however, our study slums represent majority of slum population of the city and they are the poorest……. |
| Interpretation | 20 | Give a cautious overall interpretation of results considering objectives, limitations, multiplicity of analyses, results from similar studies, and other relevant evidence | √ | 18-19 | **Strengths and limitations of this study**   - The data came from urban HDSS area, where quality of data is high and collected through three-monthly household visits by the female Field Worker. - The birth cohort was followed prospectively for neonatal survival**.** - The birth record contains socio-demographic, mode of delivery, maternity care and gestation age data. - The data is limited to selected slums (more stable one) and didn’t include vulnerable slums (side of rail line); however, our study slums represent majority of stable slums of Dhaka (North & South) and Gazipur City Corporations. |
| Generalisability | 21 | Discuss the generalisability (external validity) of the study results | √ | 18 | ……………..our study slums represent majority of slum population of the city and they are the poorest section of the rural area migrated to city for better livelihood. So, the findings of the study can be generalized for the urban poor. |
| Other information | | |  |  |  |
| Funding | 22 | Give the source of funding and the role of the funders for the present study and, if applicable, for the original study on which the present article is based | √ | 20 | Funding: This work was funded by UNICEF (Grant # 01713). |

NA: Not applicable (for this study); NR: Not reported (for study)

*Give information separately for exposed and unexposed groups.
